# Specificity effect in concrete/abstract semantic categorization task

**DOI:** 10.1007/s10339-025-01286-5

**Published:** 2025-09-03

**Authors:** Tommaso Lamarra, Caterina Villani, Marianna M. Bolognesi

**Affiliations:** Department of Modern Languages, Literatures, and Cultures, University of Bologna, Via Cartoleria, 5, Bologna, Italy

**Keywords:** Abstraction, Concreteness effect, Specificity effect, Lexical decision task, Semantic decision task

## Abstract

Concrete concepts (*banana*) are processed faster and more accurately than abstract ones (*belief*). This phenomenon, supported by empirical studies, is known as the concreteness effect. However, recent research indicates that controlling certain psycholinguistic variables can mitigate or reverse this effect. We introduce a previously neglected variable, namely categorical specificity, and investigate its role in lexical and semantic access, through: ratings, a lexical decision task and a semantic decision task. Our findings confirm the processing advantage of concrete over abstract concepts (concreteness effect) and reveal a similar advantage for specific over general concepts (specificity effect). We also report a non-significant interaction between the two variables. We discuss the results within the general framework of conceptual abstraction.

## Introduction

Current research on concrete and abstract concepts is evolving in the direction of unpacking the widely used notion of *conceptual concreteness* (Bolognesi and Steen 2018; Langland-Hassan et al. 2021; Borghi 2022; Löhr 2023; Reilly et al. 2025). An early clarification of the underspecified notion of concreteness was introduced by Connell and Lynott, through the differentiation of different perceptibility dimensions (Connell and Lynott 2012). A concept that scores high in concreteness often denotes a referent that can be perceived through the five senses (vision, touch, hearing, olfaction, and taste), such as *banana* or *book*. Conversely, concepts that score low in concreteness, namely abstract concepts, typically denote something not available in a spatiotemporal dimension and something we cannot directly perceive through our senses, such as *belief* or *bond*. Both concrete and abstract concepts result from abstraction processes, in which “knowledge of a specific category has been abstracted out of the buzzing and blooming confusion of experience” (Barsalou 2005:389). Nevertheless, there is evidence that concrete concepts and abstract concepts are processed in different ways. Typically, words denoting concrete concepts are, on average, acquired earlier (Della Rosa et al. 2010) and are more easily imageable (Paivio 1990) than abstract concepts. Moreover, concrete concepts are more tightly associated with perceptual aspects of meaning and less with linguistic ones, for their acquisition and use (Borghi 2022). This is reflected in a behavioral advantage known as the *concreteness effect*, namely the processing advantage of concrete words over abstract words. Concrete words are recognized faster and more accurately in a variety of tasks, such as lexical decision, word naming, and recall (Kounios and Holcomb 1994; Jessen et al. 2000; Romani et al. 2008). This effect is considered a cornerstone in cognitive, psychological, and linguistic studies about word processing and it can be explained by two different theories: the dual-coding theory (DCT, Paivio 1991) and the contextual availability theory (CAT, Schwanenflugel and Schoben, 1983; Schwanenflugel et al. 1988). The DCT suggests that word meaning is represented in two interconnected systems: a verbal system for linguistic information and a non-verbal system for visual and sensory information. Concrete concepts would activate both representational systems, while abstract concepts rely more on the verbal system and can only be indirectly represented in the visual system. The DCT argues that the asymmetry in the representation of concrete and abstract concepts explains the concreteness effect. On the other hand, the CAT posits that the difference between concrete and abstract concepts lies in the degree of accessibility of meaning stored in the semantic memory, rather than in the representational code. Abstract concepts, associated with multiple contexts, require more effort to retrieve from memory, while concrete concepts are more easily retrieved because they are strongly linked to a limited number of contexts.

Numerous studies have investigated processing differences between abstract and concrete concepts at both behavioral and neural levels within these theoretical frameworks, yet the findings remain inconsistent. While the empirical evidence tends overall to support the concreteness effect, some psycholinguistic studies have reported challenges in replicating it (Barca et al. 2002; Papagno et al. 2007). In certain cases, abstract concepts have even been found to have an advantage over concrete ones. This pattern has emerged in two studies employing lexical decision tasks, in which the stimuli were controlled for imageability and contextual availability, which are the two psycholinguistic variables associated with dual-coding theory (DCT) and contextual availability theory (CAT), respectively (Kousta et al. 2011; Barber et al. 2013). These results suggest that empirical studies reporting a concreteness effect did not isolate concreteness completely, leaving other variables as potential confounds for the facilitatory effect attributed to concreteness.

The current study focuses on a variable, *categorical specificity*, which shows a positive but mild/medium correlation with concreteness (Bolognesi et al. 2020; Bolognesi and Caselli 2023). Categorical specificity measures category inclusiveness, or, in classic semantics and logics, the extension (denotation) of applicability of a linguistic label to referents in the world (Ostertag 2020). Categorical specificity is a relational property that establishes taxonomical relationships between concepts and somehow defines the position of a concept within a taxonomical hierarchy: *vehicle* is a general and concrete concept, which includes more specific concrete concepts like *car* and at a subordinate level *electric car*. *Quality* is a general and abstract concept, encompassing more specific abstract concepts such as *beauty* and *elegance* (for a semantic analysis see Villani et al. 2024). Most studies on conceptual taxonomy (e.g., Rosch 1978; Rosch et al. 1976) have primarily focused on concrete concepts (e.g., “animals”, “vehicles”, “objects”), overlooking the taxonomic levels of abstract concepts. Additionally, while the basic-level advantage in object categorization is a robust effect (Murphy and Smith 1982; Jolicoeur et al. 1984; Wisniewski and Murphy 1989), its underlying sensorimotor and linguistic mechanisms remain controversial, and their implications for abstract knowledge have not yet been addressed (see von Hoef et al. 2023; Wingfiel et al. 2024). Recent research has demonstrated that categorical specificity explains a fraction of decision latencies in lexical and semantic tasks (e.g., Iliev and Axelrod 2017; Bolognesi and Caselli 2023; Ravelli et al. 2024), above and beyond conceptual concreteness. In these studies, however, the direction of the effect is unclear. Iliev and Axelrod focus on a dataset of decision latencies collected for 40,481 English words (Balota et al. 2007) and use specificity scores extracted from Wordnet. They report that highly specific words are processed more slowly than highly general words. Conversely, Bolognesi et al., as well as Ravelli et al., focus respectively on the Italian and the English ANEW datasets (Bradley and Lang 1999) and find the opposite pattern: highly specific words are processed faster than highly general words. Given these inconsistent results, the jury is still out about the nature and direction of the specificity effect, and this may be due to the different ways in which specificity is operationalized, on the nature of the task, and on the kind of words used in the studies, as further described in the Theoretical Background section of the current paper.

The present paper aims at clarifying these inconsistent findings, investigating the relationship between concreteness and specificity in two distinct processing tasks: a lexical recognition task and a semantic abstract/concrete categorization task (Pexman et al. 2017). This investigation involves a controlled set of words that were carefully selected and normed, displaying substantial variations in both concreteness and specificity.

The overarching goal of this paper is to provide additional evidence demonstrating that specificity is not merely a variable synonymous with concreteness but is, in fact, a theoretically distinct variable that maintains an intriguing relationship with concreteness. To achieve this objective, participants’ reaction times (RTs) were collected, and processing latencies were analyzed. To the best of our knowledge, no prior research has examined the interaction between word concreteness and word specificity systematically balanced within the same taxonomic relation in behavioral experiments. This is crucial for understanding how categorical specificity forms a distinct yet connected dimension with concreteness in semantic memory. Methodologically, this approach may help to disentangle previous findings on conceptual processing that could be misattributed solely to the concreteness effect. The present paper presents findings from three studies: a rating study, a lexical decision task, and a semantic decision task, all utilizing words that vary in both concreteness and specificity as stimuli. Each study addresses a specific research question, hereby summarized:

- *Study 1* A rating task where participants were asked to judge concreteness, imageability, and specificity of a set of stimuli. These judgments were used as norming study to evaluate the goodness of the stimuli, and they were factored in the analyses reported in Study 2 and Study 3.
  - *RQ1* To what extent do the theoretical distinctions introduced by analysts in stimulus design, specifically, the contrasts between concrete and abstract, and between specific and general, correspond with speakers’ judgments?
  - *Hp1* Encyclopedic knowledge and analysts’ stimuli design are expected to reflect the speakers’ knowledge. Accordingly, we hypothesize that speakers’ judgments will consistently align with the categorical distinctions between concrete and abstract, as well as general and specific concepts.
- *Study 2* A lexical decision task was employed to examine the influence of concreteness and specificity on lexical access. In this task, participants were asked to decide whether a string of letters presented on the screen was an Italian word or a nonword, following the procedure outlined by Meyer and Schvaneveldt (1971).
  - *RQ2* Do concreteness and specificity affect lexical decision latencies alone and in interaction?
  - *Hp2* We hypothesize that higher levels of concreteness will be associated with faster lexical decision latencies, consistent with the well-established concreteness effect. We also expect that greater specificity will predict faster processing times, as more specific concepts tend to be more closely linked to semantically similar contexts (Rambelli and Bolognesi 2023). In contrast, more general concepts, which are associated with a broader and more variable range of contexts, are expected to result in slower responses. This hypothesis builds on contextual availability theory (CAT), extending it with recent findings that emphasize the role of specificity in modulating contextual accessibility. Therefore, both concreteness and specificity are anticipated to have independent facilitative effects on lexical decision performance.
- *Study 3* A semantic decision task for abstract/concrete categorization (Pexman et al. 2017), requiring participants to determine whether an Italian word displayed on the screen denotes an abstract or a concrete concept.
  - *RQ3* Do concreteness and specificity affect semantic decision latencies alone and in interaction?
  - *Hp3* We expect to observe a similar pattern of results as in the LDT: concrete concepts undergoing faster processing than abstract ones. Likewise, we predict that general words will undergo slower processing compared to specific ones. Given the deeper semantic involvement in this case, the effects may be more pronounced than those observed in the LDT.

## Theoretical background

According to scientific literature, abstract and concrete concepts differ across multiple dimensions. These distinctions include the quality and quantity of information they convey (Vigliocco et al. 2009), the processes involved in their acquisition (Villani et al. 2021), their dependence on social interaction (Villani et al. 2022; Fini et al. 2023; Mazzuca et al. 2025), and the type and richness of linguistic and perceptual experiences they evoke (Lynott et al. 2020). These differences may help explain why, on average, concrete concepts are acquired earlier (Schwanenflugel 2013; Borghi 2022), are more easily recalled (Romani et al. 2008), and are processed more quickly (Schwanenflugel and Shoben 1983; Schwanenflugel et al. 1988) than abstract concepts.

Recent studies, however, report reversed effects, demonstrating a processing advantage of abstract over concrete words when various psycholinguistic variables are controlled (Kousta et al. 2011; Barber et al. 2013). For instance, the concreteness effect disappears in lexical decision latencies when words are controlled for contextual availability, suggesting that contextual availability, rather than concreteness, should be considered as the factor influencing latencies (Schwanenflugel et al. 1988, 1992).

Kousta et al. (2011) and Vigliocco et al. (2014) reported a reversed effect when concrete and abstract concepts are matched on other psycholinguistic variables: abstract concepts exhibited a processing advantage. This phenomenon may be linked to the stronger affective associations typically found in abstract concepts compared to concrete ones. However, more recent findings suggest that stronger emotion-related content is actually associated with concrete rather than abstract words (Winter 2023).

In general, the concreteness effect seems to be a topic with unresolved aspects, and alternative explanations offer additional insights into this phenomenon. An intriguing approach to exploring this subject involves shifting the focus from a singular trait or variable, like concreteness, to various aspects that may collectively contribute to generating a potential facilitatory effect.

To address this issue, researchers have proposed various hypotheses and developed alternative rating systems to better understand the information underlying concreteness ratings. One prominent approach involves collecting modality-specific norms of perceptual strength, which assess the degree to which a concept is associated with each of the five basic senses: vision, touch, hearing, smell, and taste. Studies have shown that these norms more accurately reflect the perceptual characteristics of words and concepts and offer stronger predictive power for lexical decision latencies than traditional concreteness ratings. Concreteness ratings, in contrast, tend to be biased toward the visual modality and appear to encompass more than just perceptual information (Connell and Lynott 2012).

Another method for unpacking concreteness ratings involves collecting scores on new variables and assessing their ability to explain the variance in concreteness ratings. For example, Troche et al. (2017) argue that various cognitive and perceptual dimensions (e.g., emotion, time, space, color, size, visual form) contribute to forming a conceptual topography that accounts for variability in concreteness. Davis and Yee (2023) demonstrate that approximately 50% of the variance in concreteness ratings is explained by the time and physical space required to perceive a concept. Moreover, Langland-Hassan and Davis (2023) and Langland-Hassan et al. (2021) introduced the concept of *Trial Concreteness* as an alternative, context-sensitive measure of conceptual abstractness. This approach moves beyond traditional, fixed classifications of abstract and concrete words measured in isolation, by focusing on the degree of concreteness required within specific tasks. In their paradigm, participants complete non-verbal semantic matching trials in which they are asked to identify the item that is most semantically related to a target image choosing from several alternatives. The difficulty of each trial, and thus its level of Trial Concreteness, is determined by two main factors: the visual similarity between the target and the correct choice, and how frequently those items are encountered together in everyday contexts. The approach builds on the notions of *low-dimensionality* and *high-dimensionality* (Lupyan & Mirman 2013)*.* A category is *low-dimensional* if it gathers exemplars that share few salient features, while it is *high-dimensional* if it gathers exemplars that share many salient features. Similarly, a trial has low Concreteness when “the target and correct choice items were (on average) judged neither to be found together commonly nor to be visually similar, relative to one or more of the other available choices for the trial” (Langland Hassan et al., 2021:8), while a trial has a high Concreteness if the association between the target item and the correct item are judged to be highly visually similar and commonly associable. In the context provided by competitive items, if target and correct choice items share few salient features, it is required more effort to associate them by virtue of semantic information. Conversely, if the target item and the correct item share many salient characteristics, the effort to abstract from the competitive distractors to establish the correct semantic association is smaller. The notion of low/high dimensionality emphasizes the process through which the identification of a concept requires an abstraction from exemplars or instantiation provided.

To the best of our knowledge, three studies (Iliev and Axelrod 2017; Bolognesi and Caselli 2023; Ravelli et al. 2024) have analyzed decision latencies in relation to both specificity and concreteness, finding significant effects. The authors of these studies claimed the theoretical disjunction between these two variables.

Iliev and Axelrod (2017) present a framework in which the degree of specificity within a taxonomic category reflects its informational precision. In this view, more specific categories carry more informational content. Drawing on concreteness ratings from Brysbaert et al. (2014), the authors introduce two measures of lexical precision based on WordNet (Miller 1995): semantic inclusiveness (P-inclusiveness) and semantic depth (P-depth). Semantic inclusiveness captures a word’s generality by measuring how many subordinate nodes it has in a taxonomic tree, with a larger number indicating greater generality and lower precision. Semantic depth, on the other hand, measures how far a word is from the top-level node in the taxonomy, with greater depth suggesting higher specificity.

Using these measures, Iliev and Axelrod examined how concreteness and precision predict word recognition latencies from the Balota et al. (2007) dataset, controlling for word length and frequency. Their results show that concreteness and precision exert opposite effects: more concrete words are processed more quickly, while more precise (i.e., specific) words lead to slower processing times. They interpret this pattern by suggesting that concrete words are easier to identify because they are more readily defined. In contrast, although precise words have well-defined meanings, their lower generality demands more cognitive effort to process.

However, the authors caution that their precision measures are derived from WordNet, a resource built on extensive expert knowledge, which may not reflect the lexical organization or semantic distinctions accessible to average speakers. In contrast, concreteness ratings are based on direct speaker judgments, providing a more realistic approximation of everyday conceptual understanding.

Bolognesi and Caselli (2023) propose a different approach, stressing the importance and the need for human-generated judgement of specificity, to avoid potential biases that may have impacted Iliev and Axelrod’s findings. The authors collected specificity ratings adopting a Best–Worst Scaling method (BWS, Louviere et al. 2015), which considers this variable’s relational nature. Specificity judgments using BWS have been collected for the Italian words contained in ANEW dataset (Montefinese et al. 2014) and used in statistical models to explain the variance in reaction times, available for the same set of words, in a LDT (Vergallito et al. 2020). The authors report that specificity explains a significant fraction of the variance in decision latencies, with a positive effect: the more specific a word is, the more time is required to be processed in LDT, in line with Iliev and Axelrod’s previous findings.

Finally, in a more recent study, Ravelli et al. (2024) collected specificity ratings for the English version of the ANEW dataset, using the same methods adopted by Bolognesi and Caselli (2023), namely the BWS method. Due to the higher availability of datasets of decision latencies in English compared to Italian, Ravelli and colleagues ran various analyses to investigate the role of specificity in lexical and semantic access based on different datasets of English decision latencies. Overall, the authors found that specificity explains a higher portion of variance in responding to *semantic* decision tasks (using the dataset by Pexman et al. 2017) compared to *lexical* decision tasks (datasets by Balota et al. 2007; Keuleers et al. 2012; Hutchison et al. 2013; Tucker et al. 2019; Mandera et al. 2020). Interestingly, in *semantic* decisions, specificity shows a negative effect (more specific words are processed faster than more general ones), while in *lexical* decisions, specificity does not always play a significant role (nor does concreteness), but when it does, specificity slows down reaction times, in line with previous findings. The differing patterns observed across task types, specifically, lexical decision tasks (LDT) and semantic decision tasks (SDT), suggest that these tasks engage distinct cognitive processes. This highlights the need for further investigation into the nature and behavior of the specificity effect. Additionally, the ANEW dataset used by Ravelli et al. (2024) consists of words selected for their high affective content, which may limit the generalizability of the findings. A more systematic examination of specificity effects on word processing is therefore warranted, using stimuli that are carefully controlled and span a range of conceptual categories across multiple taxonomic levels.

The current study builds on these previous findings and arguments. Since effects and results of Lexical Decision Task and Semantic Decision Task seem to diverge, suggesting that different aspects of language are recognized and gathered with these kinds of tasks (van Hees et al. 2016; Pexman et al. 2017), we decided to collect data both of lexical and semantic access to meaning in words that vary in concreteness and specificity. We based our analyses on a set of stimuli carefully constructed to include words systematically high and low in specificity and high and low in concreteness.

This setup allows us to carefully examine the distinct but integrated aspects involved in the conceptual abstraction process: the extent to which the referent designated by a linguistic term can be perceived through sensory experience (concreteness), and the degree of extension or precision of a conceptual category (specificity) (see Reilly et al. 2025). When speakers engage in lexical or semantic decision tasks involving relatively more abstract or concrete concepts, their performance may be influenced by the cognitive effort required to abstract a referent from a single or multiple category exemplars (e.g., Nosofsky 1986; Daelemans and van den Bosch 2005; Chandler 2017). Consequently, the concreteness effect may interact with word specificity, resulting in a processing advantage for concepts that are both highly concrete and specific, as well as for highly abstract but specific concepts. Conversely, there may be a disadvantage for concepts low in both concreteness and specificity. If there were no interaction between concreteness and specificity, a similar pattern of results would be expected, but this would suggest an additive effect of the two variables on conceptual processing, further reinforcing the idea that concreteness and specificity are independent factors in the abstraction process (Bolognesi et al. 2020).

## The present studies

Study 1 consists of a rating task in which participants evaluate the imageability, concreteness, and specificity of the stimuli. This step serves to verify that the stimuli, as designed by the researchers, are perceived by average speakers as differing along these three dimensions. Study 2 involves a lexical decision task (LDT) conducted using the same set of stimuli. Study 3 is a semantic decision task (SDT) in which participants are asked to judge whether each word refers to a concrete or an abstract entity. This type of semantic decision task is grounded in the theoretical framework outlined by the Calgary Semantic Decision Project (CSDP; Pexman et al. 2017). According to this framework, LDT and SDT afford different processing mechanisms because word processing unfolds through an interactive and cascading sequence of processes, as argued in earlier work (Balota 1990; Yapet al. 2015). Specifically, orthographic input is initially processed at the letter level, followed by processing at the lexical level, which involves structural and formal properties of the word, such as morphosyntactic features and frequency. Semantic processing constitutes an additional level, where conceptual meaning is activated. Information flows bidirectionally between these levels (interactive activation) and does so continuously, without requiring the completion of one level before the next is engaged (cascading activation). Both lexical and semantic information can influence performance in word processing tasks, although their relative contributions may vary depending on task-specific demands.

Data analysis and visualization of all the studies were carried out with R (RCore Team 2023) and RStudio (v. 4.2.3). Ratings scores and Reaction Times (RTs) were modeled with linear mixed models using lmer() function form “lme4” R’s package (Bates et al. 2015). Post-hoc contrasts on rating scores were carried out with “emmeans” R’s package (Lenth 2021) using Tukey’s adjustment for multiple comparisons.

All the experiments reported here were conducted with the Ethical approval of the Ethics Committee of the University of Bologna (Protocol n. 0121435). All participants received guidelines and instructions as well as informed consent. All participants agreed to participate in the experiments voluntarily. 

Data and analyses scripts of the three studies are publicly available in the following Open Science Framework repository: https://osf.io/j2smv/

## Study 1: ratings

### Method

#### Participants

A total of 90 native Italian speakers participated in the study. Participants for the concreteness and imageability rating tasks were recruited via the Prolific crowdsourcing platform (https://www.prolific.com/), and the tasks were administered using Qualtrics survey software (https://www.qualtrics.com/). For the specificity rating task, participants were recruited through Qualtrics by distributing the survey among colleagues from the Department of Modern Languages, Literatures and Cultures at the University of Bologna, as well as through personal networks. Thirty participants (18 female, 12 males; *M*_age_ = 32.1, *SD*_age_ = 7.72; 83.3% had a Master’s Degree, 13.3% had a Bachelor’s Degree, 3.33% had a PhD) completed the concreteness rating task; 30 participants (18 female, 12 males; *M*_age_ = 33.9, *SD*_age_ = 9.44; 73.3% had a Master’s Degree, 10% had a Bachelor’s Degree, 16.7% had a PhD) completed the imageability rating task and 30 participants (19 female, 11 males; *M*_age_ = 31.6, *SD*_age_ = 9.26; 56.7% had a Master’s Degree, 16.7% completed High School, 13.4% had a Bachelor’s Degree, 13.3% had a PhD) completed the specificity rating task. All participants were naïve as to the purpose of the experiment.

#### Materialsnull

The stimuli consisted of 120 words selected and adapted from the materials of a previous study (Villani et al. 2022). This dataset includes 30 abstract concepts and 30 concrete concepts, each distributed across three semantic domains. For concrete concepts, the domains were “Animal” (*n *= 10), “Food” (*n* = 10), ad “Tools” (*n* = 10). For abstract cocepts, the domains were “PSTQ” (physical–spatio-temporal–quantitative;* n* = 10), “PS” (philosophical–spiritual; *n* = 10), and “EMS” (emotional–moral–social; *n* = 10). We chose to differentiate abstract and concrete concepts into subcategories to allow for greater granularity in the analysis. However, a detailed investigation of distinctions among these subcategories and their levels of specificity falls outside the scope of the present study.

For each concept, we generated both a hypernym and a hyponym. The identification and evaluation of specificity levels were primarily conducted using WordNet (MultiWordNet: https://multiwordnet.fbk.eu). In cases where WordNet did not provide the necessary lexical entries due to gaps in the resource, we consulted the Treccani Italian dictionary (https://www.treccani.it/enciclopedia/dizionario), selecting terms explicitly linked by an IS-A relationship to a plausible hypernym or hyponym. Moreover, when the input item was already fairly general or fairly specific, we kept the original stimulus (e.g., “insect”). The final set of stimuli used in this study consisted of 30 specific-concrete concepts (e.g., *dromedary*), 30 general-concrete concepts (e.g., *mammal*), 30 specific-abstract concepts (e.g., *euphoria*), and 30 general-abstract concepts (e.g., *disquiet*).

We balanced the stimuli for word length and word frequency computed from he corpus itTenTen20 using SketchEngine (Jakubíček et al. 2013). We performed one-way analyses of variance (ANOVA) in R using the aov() function. Results showed that the four subgroups of concepts did not differ in terms of number of letters (*F*(3,116) = 0.357, *p* = 0.784) and (log) frequency (*F*(3, 116) = 0.506, *p* = 0.679). The mean and standard deviation for length and frequency of stimuli are shown in Table 1.

**Table 1:** Descriptive statistics of four classes of concepts used as stimuli in the experiments

Concepts	Frequency		Length	
	Mean	SD	Mean	SD
Abstract-general	8.30	1.88	5.09	0.50
Abstract-specific	8.13	2.27	5.02	0.73
Concrete-general	8.03	2.54	5.01	0.75
Concrete-specific	7.73	1.98	4.89	0.42

#### Procedure

The study was implemented as three distinct online questionnaires on Qualtrics, each administered to a separate group of participants. Following the consent form, a demographic survey, and instructions, participants rated 120 words using a 5-point Likert scale for one of the following variables:

- Concreteness: Participants rated how much each word referred to something “concrete,” which could be perceived through the five senses, or something “abstract,” which could hardly be perceived through the five senses, from 1 = very abstract to 5 = very concrete.
- Imageability: Participants rated how easy it was to associate each word with a mental image (e.g., a scene, a situation, or another sensory experience), from 1 = difficult to imagine to 5 = easy to image.
- Specificity: Participants rated how much a word refers to something very specific, from 1 = not at all specific (i.e., concepts referring to a wider group of objects/entities or situations) to 5 = very specific (i.e., concepts referring to something precise).

We operationalized both concreteness and imageability, as previous studies have suggested that imageability may be a stronger predictor of lexical decision latencies than concreteness (Vergallito et al. 2020).

In each survey, the word stimuli were randomly presented in two blocks of 60 items, and participants completed a set of practice trials before beginning the main task. The full instructions provided to participants are available in the OSF repository. Data collection continued until 30 ratings were obtained for each variable.

### Data analyses and results

We ran three separate linear mixed models using rating scores (i.e., concreteness, imageability, specificity) as dependent variable and concept type (i.e., abstract-general, abstract-specific, concrete-general, concrete-specific) as independent variable, including participants and concepts as random intercepts.

Table 2 shows descriptive statistics for ratings scores by four quadrants. Results revealed that there is an overall effect of the type of concept labelled by analysts on the concreteness ratings judged by participants (*F*(3, 116) = 345.47, *p* < 0.001), imageability (*F*(3, 116) = 167.67, *p* < 0.001.), and specificity ratings (*F*(3, 116) = 85.821, *p* < 0.001). Post-hoc contrasts showed significant differences in concreteness and in imageability, between abstract-general and concrete-general (*p*_*s*_ <. 0.001), as well as between abstract-specific and concrete-specific (*p*_*s*_ <. 0.001), as expected. Moreover, there were no significant differences in concreteness and imageability between abstract-general and abstract-specific (*p* = 0.44, *p* = 0.60; respectively), as expected. However, we found unexpected significant differences in concreteness and imageability between concrete-general and concrete-specific (*p* = 0.0002, *p* = 0.0006; respectively). Considering the specificity ratings, we found an unexpected significant difference in specificity between abstract-specific and concrete-specific concepts (*p* < 0.001), as well as between abstract-general and concrete-general (*p* = 0.031). Finally, we computed Pearson’s correlation between the three variables. As expected, we found a high positive correlation between concreteness and imageability (*r* = 0.96), a low positive correlation between specificity and concreteness (*r* = 0.29) and between specificity and imageability (*r* = 0.35).

**Table 2:** Mean and standard deviation for concreteness, imageability, and specificity ratings by four quadrants

	Concreteness		Imageability		Specificity	
	Mean	SD	Mean	SD	Mean	SD
Abstract-general	1.78	0.988	2.23	1.16	2.9	1.30
Abstract-specific	1.97	1.18	2.41	1.25	3.43	1.25
Concrete-general	4.46	0.819	4.30	0.957	2.55	1.23
Concrete-specific	4.99	0.185	4.86	0.488	4.48	0.868

### Discussion

Our findings reveal a pattern that aligns with previous research. We observed a modest but consistent positive correlation between specificity and concreteness, in line with the results reported by Bolognesi et al. (2020). In contrast, concreteness and imageability exhibited a much stronger correlation, as documented in earlier studies by Richardson (1976), Connell and Lynott (2012), and Khanna and Cortese (2021). Given this high correlation between imageability and concreteness, and following an evaluation of potential collinearity (see below), we decided to retain only concreteness and specificity as predictors in the subsequent studies.

The results from this rating study also offer insight into the internal structure of the stimuli. Within the set of concrete concepts, we found a significant difference in concreteness ratings between general items, such as *"essere vivente"* (*living being*), and specific items, such as *"pastore tedesco"* (*German shepherd*). However, within the set of abstract concepts, no significant difference in concreteness was observed between general concepts, such as *"attributo"* (*attribute*), and specific concepts, such as *"angoscia"* (*anguish*). We interpret this pattern as evidence that the relationship between specificity and concreteness is more complex in the domain of concrete concepts. In contrast, in the domain of abstract concepts, the two variables appear to function independently.

This distinction may be explained by differences in referential structure. General concepts tend to refer to categories that include many potential members, while specific concepts refer to a smaller, more narrowly defined set. The broader potential for instantiating general concepts may enhance their perceived concreteness. For example, a concept like *substance* may evoke a wider range of concrete exemplars than a more specific term like *Aspirin*. In abstract concepts, such differences are less likely to emerge because both general and specific abstract terms typically lack a clear, tangible referent.

The speaker-generated ratings collected in this first study were used as predictors in Study 2, which involved a lexical decision task, and Study 3, which involved a semantic decision task. These ratings were chosen in place of the analyst-defined categorical distinctions used during stimulus construction, as they more closely reflect participants’ conceptual knowledge and offer greater ecological validity for understanding how words are processed.

## Study 2: Lexical decision task

### Method

#### Sample size rationale

Based on a review of previous research, we recruited 43 and 44 participants for the two behavioral experiments, respectively. This exceeds the sample sizes typically employed in comparable lexical decision task studies, such as Barber et al. (2013, *n* = 18), Sidhu et al. (2016, *n* = 30), and Pauligk et al. (2019, *n* = 30).

#### Participants

43 students (32 female; 11 males; *M*_age_ = 22.9; *SD age* = 3.14) of the University of Bologna took part in the study as volunteers. All participants were native Italian speakers and were naïve as to the purpose of the experiment.

#### Materials

We used as stimuli the 120 Italian words described in Study 1, along with 120 nonwords generated using an online tool (https://www.trainingcognitivo.it/GC/nonparole/). The nonwords were selected to match the real words in terms of syllable count and overall length.

#### Procedure

Participants were instructed to determine whether each presented stimulus was a real word or a nonword.

Each participant was tested individually in a quiet environment at the Experimental Lab, Department of Modern Languages, Literatures and Cultures, University of Bologna. The experiment was conducted on a PC using E-Prime 3.0 software (Psychology Software Tools, Pittsburgh, PA). Stimuli were presented in uppercase Calibri font, size 32, in white text on a black background, centered on the screen. Participants were seated comfortably at a distance of approximately 60 cm from the monitor. The experiment consisted of 240 trials, comprising two blocks of 60 words and 60 nonwords each.

Each list included an equal number of general-concrete words (5 foods, 5 tools, 5 animals); specific-concrete words (5 foods, 5 tools, 5 animals), general-abstract words (5 PSTQ, 5 PS, 5 EMS), specific-abstract (5 PSTQ, 5 PS, 5 EMS). Stimuli were presented in random order in the four blocks. Each block was composed of 30 nonwords and 30 words, including 15 abstract concepts and 15 concrete concepts. The blocks were designed to ensure that specific and general concepts belonging to the same taxonomic relation did not appear within the same block. For example, given the basic concept *banana*, the specific term *plantain* was assigned to one block, while the more general term *fruit* was placed in the other. Each trial began with a fixation cross displayed at the center of the screen for 1000 ms, followed by a blank screen for 500 ms. After the blank screen, the target letter strings appeared and remained on the screen for 500 ms. Participants had 1000 ms to categorize each stimulus as a word or a nonword by pressing the left (“x”) or right (“m”) key on the keyboard. The assignment of response keys was counterbalanced across participants to minimize response bias. Participants were instructed to respond as quickly and accurately as possible.

To minimize semantic priming effects, participants were given a short break every 60 trials and a 10-min break after completing the first two blocks, during which they performed simple arithmetic calculations before continuing the task. Before beginning the LDT, participants received practice trials consisting of four words and four nonwords. Each behavioral session lasted approximately 20 min, including the break between blocks.

### Data analysis and results

Practice trials, nonword trials, and errors (4.8%) were excluded from the analysis. All the remaining participants exhibited an accuracy rate greater than 80%. Consequently, we retained all participants for subsequent analyses. Furthermore, we excluded from analyses 3 items (i.e., “prodotto agricolo” *agricultural product*; “elisio” *elysium*, “dromedario” *dromedary*) with an accuracy rate lower than 70%.

We used linear mixed effects models to analyze the data. Initially, we modeled RTs as a function of the three variables (i.e., concreteness, imageability, specificity), with participants and number of concepts as random intercepts. To assess collinearity among predictor variables in the regression, variance inflation factors (VIF) (Fox 2005) were calculated using vif() function form “car” R’s package. The VIF values indicated varying degrees of collinearity, with concreteness and imageability having VIF values exceeding 10 (11.96 and 12.57, respectively), while specificity showed a lower VIF (1.20). Given the higher VIF value for imageability, we chose to exclude this variable from subsequent models. However, in the interest of transparency, analyses including both imageability and specificity as predictors are available in the OSF repository.

As previously noted, all models included participants and the number of concepts as random intercepts. We employed a stepwise modeling approach. First, we specified a baseline model with only random effects (Model 0). We then introduced concreteness as a fixed effect (Model 1), followed by a model including only specificity as the predictor (Model 2). Next, we examined the combined influence of concreteness and specificity (Model 3), and finally, we tested their interaction (Model 4). This sequential approach enabled a systematic evaluation of the individual and joint contributions of concreteness and specificity to reaction times.

The following scripts summarize the 4 analyses:

- Model 0: *lmer (RT* ~ *(1| Subject)* + *(1| concepts)*
- Model 1: *lmer (RT* ~ *mean.con* + *(1| Subject)* + *(1| concepts)*
- Model 2:* lmer (RT* ~ *mean.spe* + *(1| Subject)* + *(1| concepts)*
- Model 3: *lmer (RT* ~ *mean.con* + *mean.spec (1| Subject)* + *(1| concepts)*
- Model 4: *lmer (RT* ~ mean.con * mean.spe + *(1| Subject)* + *(1| concepts)*

Table 3 summarizes the results of the four linear mixed models. None of the models reports a significant effect of concreteness, of specificity, the combination of concreteness and specificity or their interaction over reaction times in the LDT. However, concreteness approaches significance in explaining reaction times (*p* = 0.06). The random effects in each model suggest substantial variability at the concept and subject levels, emphasizing the importance of accounting for these factors. Bar plots showing the random effects of each concept in each model are available in the OSF repository.

**Table 3:** Results of linear mixed models for lexical decision task: estimation and significance testing of (a) fixed and (b) random effects

(a) Fixed effect						
Model	Predictor	Estimate (β)	SE	df	t-value	*p*-value
Model 0	(Intercept)	628.47	15.34	53.22	40.98	< 0.001
Model 1	(Intercept)	650.723	19.313	109.037	33.694	< 0.001
	mean.con	− 6.760	3.574	114.679	− 1.891	0.0611
Model 2	(Intercept)	631.4918	25.9922	154.1620	24.295	< 0.001
	mean.spe	− 0.9064	6.3009	114.5187	− 0.144	0.886
Model 3	(Intercept)	642.637	26.420	153.814	24.324	< 0.001
	mean.con	− 7.275	3.766	113.798	− 1.932	0.0558
	mean.spe	2.939	6.538	113.680	0.449	0.6540
Model 4	(Intercept)	603.647	74.465	121.414	8.106	< 0.001
	mean.con	2.510	17.871	112.890	0.140	0.889
	mean.spe	14.978	22.469	113.198	0.667	0.506
	mean.con:mean.spe	− 2.932	5.233	113.040	− 0.560	0.576

(b) Random effects			
Models	Group	Variance	Std.Dev
Model 0	Concepts	3167	56.27
	Subject	8832	93.98
	Residual	13,558	116.44
Model 1	Concepts	3091	55.60
	Subject	8829	93.96
	Residual	13,558	116.44
Model 2	Concepts	3196	56.54
	Subject	8832	93.98
	Residual	13,558	116.44
Model 3	Concepts	3116	55.82
	Subject	8829	93.96
	Residual	13,558	116.44
Model 4	Concepts	3137	56.00
	Subject	8829	93.96
	Residual	13,558	116.44

Numbers of observations: 4876; Concepts:117; Subjects: 43

Furthermore, a comparative analysis of model fits using the anova() function indicated that the inclusion of concreteness in Model 1 may improve model fit relative to Model 0, which included only random effects. However, the evidence supporting this improvement is limited. The subsequent addition of specificity in Model 3, as well as the interaction between concreteness and specificity in Model 4, did not lead to a significant improvement in model fit for explaining variance in reaction times. See Table 4.

**Table 4:** Lexical decision: Model comparison metrics with Concreteness added first

	AIC	BIC	logLik	Deviance	Chisq	df	Pr(> Chisq)
Model 0	60,699	60,725	− 30,346	60,691			
Model 1	60,698	60,730	− 30,344	60,688	35.580	1	0.059
Model 3	60,699	60,738	− 30,344	60,687	0.2055	1	0.65
Model 4	60,701	60,746	− 30,344	60,687	0.3220	1	0.57

Additionally, we conducted a supplementary model comparison by reversing the order of inclusion of the two main predictors: specificity was added first, followed by concreteness. The results indicated that Model 2, which included specificity in addition to the baseline Model 0, did not significantly improve model fit. Model 3, which included both specificity and concreteness, yielded only a marginal improvement. Finally, the inclusion of the interaction term in Model 4 did not result in a significant enhancement of model fit. See Table 5.

**Table 5:** Lexical decision: Model comparison metrics with Specificity added first

Model	AIC	BIC	logLik	Deviance	Chisq	Df	Pr(> Chisq)
Model0	60,699	60,725	− 30,346	60,691			
Model2	60,701	60,734	− 30,346	60,691	0.0209	1	0.885
Model3	60,699	60,738	− 30,344	60,687	3.7426	1	0.053
Model4	60,701	60,746	− 30,344	60,687	0.3220	1	0.57

### Discussion

Our findings suggest that concreteness and specificity do not substantially influence reaction times at the lexical level. This may be attributed to the fact that both concreteness and specificity are semantic in nature and are more likely to exert an effect when participants engage in deeper cognitive processing that accesses the semantic content of the stimuli. In lexical decision tasks, however, participants often rely on relatively shallow processing of word forms rather than on full semantic interpretation (Balota et al. 1991; Yap et al. 2011; Pexman et al. 2017).

Indeed, some researchers (McClelland and Rumelhart 1981; Balota et al. 1991) have proposed a model of word processing in which information flows between semantic units (or “meaning-level units”) and lexical units (or “word-level units”). In this framework, semantic units provide feedback activation to lexical units. Although semantic information can contribute to visual word recognition, its role is generally secondary to that of lexical-level properties (Yap et al. 2011; Pexman et al. 2017).

The absence of significant effects for concreteness and specificity may also be explained by the short presentation duration used in our task. This contrasts with previous studies that have reported concreteness effects using longer exposure times, such as 2000 ms in the study by Vergallito et al. (2020). Nevertheless, our exploratory models reveal a significant effect of imageability, suggesting that different semantic variables may require varying exposure durations to influence word recognition in lexical decision tasks (see OSF repository).

However, a direct comparison between our findings and previous lexical decision studies is not entirely appropriate. Many of those studies primarily examined concepts at the basic level of categorization, as seen in Barca et al. (2002), Vergallito et al. (2020), and Kousta et al. (2011), whereas our stimuli include concepts that span multiple levels of a taxonomic hierarchy. Furthermore, these studies often employed different experimental methodologies and paradigms, such as repetitive transcranial magnetic stimulation in Papagno et al. (2009), functional magnetic resonance imaging in Vigliocco et al. (2014), or response modalities involving mouth and motor key-presses in experiments with concrete, abstract, and emotional concepts, as in Mazzuca et al. (2018).

**Fig. 1:**
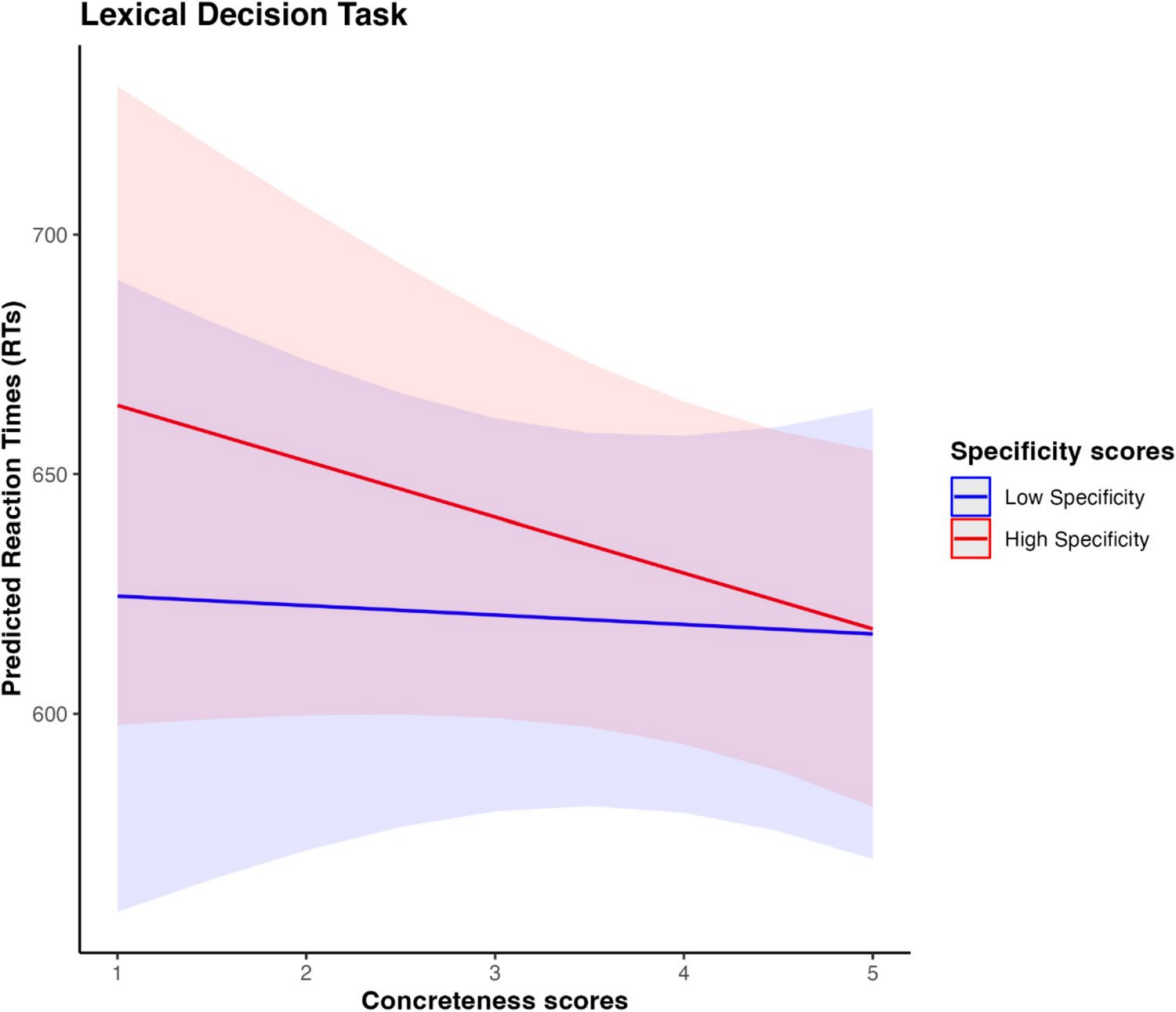
Interaction effect between Concreteness and Specificity variables in predicting RTs in Lexical Decision Task. Blue line refers to low specificity scores (i.e., general concepts) and red line refers to high specificity scores (i.e., specific concepts)

A visual inspection of the interaction between concreteness and specificity (see Fig. 1) reveals several notable trends. Among concepts with lower concreteness scores, which correspond to abstract concepts, differences in processing latencies appear more pronounced. In particular, specific abstract concepts tend to be processed more slowly than general abstract concepts. In contrast, among more concrete concepts, both general and specific items show similar processing times. Finally, highly general concepts, whether abstract or concrete, do not appear to be processed differently from one another.

## Study 3: Semantic decision task

### Method

#### Sample size rationale

In line with Study 2, our sample size consisted of at least 40 participants.

#### Participants

44 students (33 female; 11 males; *M*_age_ = 22.6; *SD*_age_ = 2.86) of the University of Bologna took part in the study as volunteers. All participants were native Italian speakers and were naïve as to the purpose of the experiment.

#### Materials

Stimuli consisted of the 120 Italian words used in the Lexical Decision Task.

#### Procedure

Participants performed a semantic decision task (SDT) in the same place and in the same conditions described in Study 2. The task consisted of a semantic decision in which they were asked to categorize whether the word on the screen denotes a concrete or an abstract concept, as in Pexman et al. (2017).

The experiment consisted of 120 trials divided into two blocks of 60 words. Each block included 30 concrete and 30 abstract concepts, varying in specificity and carefully balanced across sub-categories. To prevent semantic priming effects during the task, concepts sharing the same taxonomical relation were kept in separate blocks, as in Study 2. Before the experimental session, participants were provided with 8 practice trials including 4 abstract concepts (2 general, 2 specific) and 4 concrete concepts (2 general, 2 specific). Each experimental trial began with a fixation cross presented at the center of the screen for 1000 ms, followed by a blank screen for 500 ms. After the blank screen, the target word appeared and remained on the screen for a maximum of 3000 ms or until participant’s response. Participants had to categorize stimuli as a concrete concept or as an abstract concept by pressing the left (“x) or right (“m”) key on keyboard. The assignment of responses to keys was counterbalanced across participants to avoid any bias. Participants were instructed to respond as quickly and accurately as possible.

To reduce any semantic priming effects, participants were given a break between the two blocks, during which they performed simple arithmetic calculations. Each behavioral session lasted approximately 15 min, including the break.

### Data analysis and results

Practice trials and errors (8.47%) were excluded from the analysis. All participants achieved an accuracy greater than 80%, thus, we retained them for subsequent analyses. Moreover, we removed 8 items (i.e., “dollaro” *dollar*, “valuta” *currency*, “paga” *payment*, “diagramma” *diagram*, “copertura” *coverage*, “elisio” *elysium*, “sezione” *section*, “rivolta” *revolt*) with an accuracy rate lower than 70%, indicating that participants did not clearly classify these words as either abstract or concrete.

We used linear mixed effects models to analyze the data. Initially, we modeled RTs as function of the three variables (i.e., concreteness, imageability, specificity), with participants and number of concepts as random intercepts. To assess collinearity among predictor variables in the regression, Variance Inflation Factors (VIF) (Fox 2005) were calculated using vif() function form “car” R’s package. As in Study 2, the VIF values indicated varying degrees of collinearity, with concreteness and imageability having VIF values exceeding 10 (12.65 and 13.27, respectively), while specificity showed a lower VIF (1.19). Given the higher VIF value for imageability, we chose to exclude this variable from subsequent models. However, for sake of transparency, analysis including imageability and specificity as predictors are reported in the OSF repository.

In line with our hypotheses, we replicated the same four models from Study 2, using reaction times (RTs) as the dependent variable and rating scores as independent variables. All models incorporated participants and the number of concepts as random intercepts. Specifically, we consider random effects (model 0), followed concreteness (model 1), and specificity (model 2), then joint influence of concreteness and specificity (model 3) and lastly, their interaction (model 4) as predictors.

The following scripts summarize the 4 analyses, which mirror the models run in Study 2:

- Model 0: *lmer (RT* ~ *(1| Subject)* + *(1| concepts)*
- Model 1: *lmer (RT* ~ *mean.con* + *(1| Subject)* + *(1| concepts)*
- Model 2:* lmer (RT* ~ *mean.spe* + *(1| Subject)* + *(1| concepts)*
- Model 3: *lmer (RT* ~ *mean.con* + *mean.spec (1| Subject)* + *(1| concepts)*
- Model 4: *lmer (RT* ~ mean.con * mean.spe + *(1| Subject)* + *(1| concepts)*

**Fig. 2:**
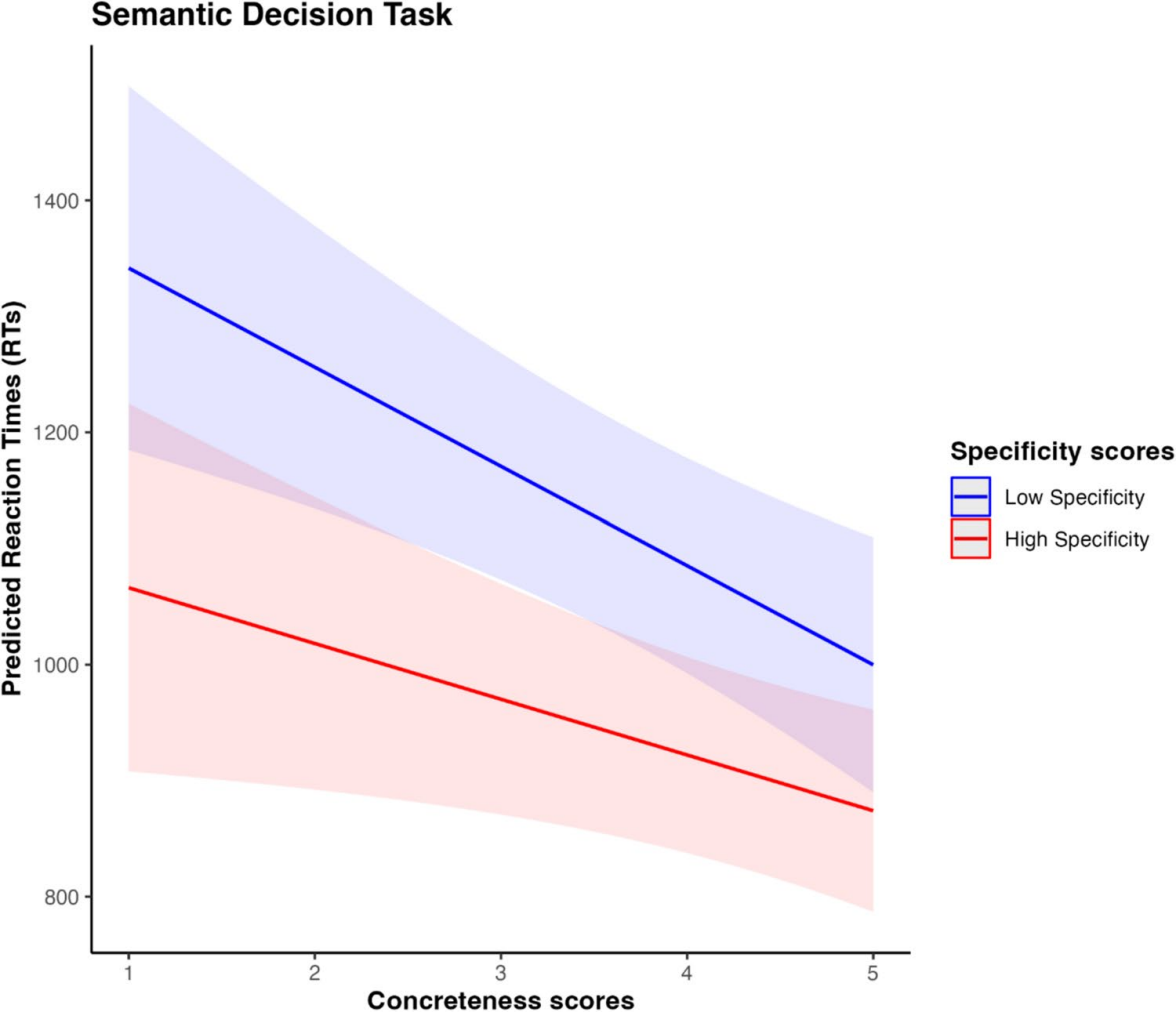
Interaction effect between Concreteness and Specificity variables in predicting RTs in Semantic Decision Task. Blue line refers to low specificity scores (i.e., general concepts) and red line refers to high specificity scores (i.e., specific concepts)

Table 6 shows results of the four linear mixed models. The results of Model 1 indicate a significant effect of word concreteness on reaction times. The result of Model 2 shows a significant effect of word specificity on the dependent variable. When both variables are introduced in Model 3, a significant effect is observed for concreteness and specificity. However, Model 4 does not yield sufficient evidence for a significant interaction between the two variables (see Fig. 2). Bar plots showing the random effects of each concept in each model are available in the OSF repository.

**Table 6:** Results of linear mixed models for semantic decision task: estimation and significance testing of (a) fixed and (b) random effects

(a) Fixed effect						
Model	Predictor	Estimate (β)	SE	df	t-value	*p*-value
Model 0	(Intercept)	1045.14	38.02	64.31	27.49	< 0.001
Model 1	(Intercept)	1287.74	46.65	114.31	27.605	< 0.001
	mean.con	− 73.22	8.76	108.35	− 8.358	< 0.001
Model 2	(Intercept)	1317.59	70.32	149.41	18.736	< 0.001
	mean.spe	− 81.60	17.82	109.15	− 4.578	< 0.001
Model 3	(Intercept)	1419.944	62.459	149.119	22.734	< 0.001
	mean.con	− 64.971	8.837	107.473	− 7.352	< 0.001
	mean.spe	− 47.783	15.322	107.554	− 3.119	0.002
Model 4	(Intercept)	1571.88	176.86	115.46	8.888	< 0.001
	mean.con	− 102.73	42.06	107.14	− 2.442	0.0162
	mean.spe	− 94.73	53.37	107.45	− 1.775	0.0787
	mean.con:mean.spe	11.32	12.33	107.11	0.918	0.3605

(b) Random effects			
Model	Group	Variance	SD
Model 0	Concepts	31,185	176.6
	Subjects	50,408	224.5
	Residual	99,182	314.9
Model 1	Concepts	18,281	135.2
	Subjects	50,413	224.5
	Residual	99,188	314.9
Model 2	Concepts	26,069	161.5
	Subjects	50,407	224.5
	Residual	99,181	314.9
Model 3	Concepts	16,772	129.5
	Subjects	50,412	224.5
	Residual	99,187	314.9
Model 4	Concepts	16,800	129.6
	Subjects	50,414	22.5
	Residual	99,186	314.9

Number of observations: 4671; Concepts: 112; Subjects: 44

A comparative analysis of models fits using anova() function showed that adding concreteness score in Model 1 significantly improves model fit compared to Model 0 with only random effects. Importantly, adding specificity scores in Model 3 significantly improves model fit compared to using only concreteness scores. In contrast, Model 4 with interaction between the two variables did not improve the model fit. See Table 7.

**Table 7:** Semantic decision: model comparison metrics with concreteness added first

	AIC	BIC	logLik	Deviance	Chisq	df	Pr(> Chisq)
Model 0	67,471	67,497	− 33,732	67,463			
Model 1	67,419	67,451	− 33,704	67,409	54.6250	1	< 0.001
Model 3	67,411	67,450	− 33,700	67,339	9.5065	1	0.002
Model 4	67,412	67,457	− 33,699	67,398	0.8645	1	0.35

In the second comparative analysis, we found that Model 2, which adds specificity to the baseline model 0 with only random effects, improves the model fit. Similarly, Model 3, which includes both specificity and concreteness, showed a significant improvement. Nevertheless, the Model 4 with the interaction term included did not produce a significant improvement. See Table 8.

**Table 8:** Semantic decision: model comparison metrics with specificity added first

Model	AIC	BIC	logLik	Deviance	Chisq	df	Pr(> Chisq)
Model 0	67,471	67,497	− 33,732	67,463			
Model 2	67,454	67,486	− 33,722	67,444	194.029	1	<.001
Model 3	67,411	67,450	− 33,700	67,399	44.7286	1	<.001
Model 4	67,412	67,457	− 33,699	67,398	0.8645	1	0.3525

### Discussion

In the semantic decision task, we replicated the concreteness effect, with higher concreteness scores associated with shorter decision latencies. Notably, we also observed a pattern that we refer to as the "specificity effect": concepts with higher specificity scores were processed more quickly than those with lower scores, namely, general concepts. Consistent with our hypothesis, general words showed slower processing times than specific words, possibly because general terms refer to a broader range of category members and are associated with more varied contexts (Rambelli and Bolognesi 2023). One possible interpretation is that more specific words activate a narrower and more focused semantic network, facilitating faster access to a core representation, whereas general words may trigger broader, less defined networks, resulting in slower processing.

Crucially, the absence of an interaction between concreteness and specificity in explaining variation in reaction times (RTs) suggests that, although the two variables show similar patterns, they contribute independently to word processing. The additive effects on decision latencies, along with the low correlation between the two variables (*r* = 0.29), support the argument that concreteness and specificity should not be conflated (Bolognesi et al. 2020). Instead, they should be considered distinct factors that reflect different dimensions of semantic representation.

A comparison between the model including both concreteness and categorical specificity and the model containing only concreteness showed that the addition of specificity substantially improved the model’s ability to predict RTs. Although the two predictors do not contribute equally, this imbalance is to be expected given the nature of the task, which requires participants to categorize words as concrete or abstract. In this context, it is unsurprising that concreteness accounts for a slightly greater proportion of variance than specificity. Importantly, the finding that specificity significantly influenced performance in a semantic decision task focused on concreteness highlights its role as an independently activated semantic variable. This suggests that specificity contributes to semantic processing in a way that is at least partially automatic and not simply a result of task-related attention or explicit instruction. This pattern is consistent with the mechanisms described by Connell and Lynott (2016), who argue that perceptual strength, as a dimension of semantic richness, supports the efficient transfer of salient information from unconscious to conscious processing, regardless of the task. In our study, specificity may function in a similar manner. Although participants were not instructed to consider specificity, the underlying cognitive structure of more or less specific concepts may have inherently influenced their categorization decisions. This interpretation adds theoretical significance to the results and emphasizes the importance of treating specificity as a fundamental component of conceptual representation, distinct from but complementary to concreteness.

## General discussion

In the present study, we investigated the role of word specificity in language processing and its impact on the widely recognized but still debated phenomenon known as “concreteness effect”, i.e., the processing advantage of concrete over abstract concepts. As anticipated in the introduction of this work, categorical specificity is a variable often neglected and sometimes confused with concreteness. Recent studies have provided evidence of a positive, though mild, correlation between these two variables (Bolognesi and Caselli 2023). Both concrete and abstract concepts can define highly general and inclusive conceptual categories (e.g., *anima*l, *feeling*) or highly specific and constrained conceptual categories (e.g., *pitbull*, *enthusiasm*). However, since existing studies working on differences between concrete and abstract concepts did not control stimuli for specificity, it remains an open question whether the level of specificity may contribute to the concreteness effect, introducing potential confounding evidence reported in the literature. Consistent with this, recent studies reviewed in the present article have shown that word specificity accounts for a significant portion of variance in response latencies during word processing. Nevertheless, those studies reported different directions of such effect, with specific words being processed either faster or slower than general ones. This inconsistency may stem from the diverse criteria used in selecting linguistic stimuli (from WordNet or human-generated data), and the nature of the task performed.

The main aim of our work is to shed light on the direction of specificity effect during language processing and its role in explaining “concreteness effect”. To achieve this goal, we conducted three studies: a norming study and two decision tasks. Specifically, we performed a lexical decision and a semantic categorization task, using a controlled set of words varying in concreteness and specificity (i.e., abstract-specific, abstract-general, concrete-specific, concrete-general) and balanced for word length and frequency. Reaction times for each task were modeled as function of specificity and concreteness ratings elicited by speakers during a norming study on our stimuli.

Our findings support and extend previous research on word specificity and word concreteness. The results of Study 1 confirm that when speakers were asked to judge word specificity both abstract and concrete concepts were characterized by different levels of specificity even though the differences in specificity are less pronounced for abstract than for concrete concepts, in line with what is argued by Borghi (2022). Moreover, norming data show the asymmetry between the knowledge derived by human speakers and that extracted by a dictionary-based resource, such as WordNet. While the latter can be a valuable tool to capture hierarchical taxonomic relations, it does not accurately reflect speakers’ lexical knowledge and organization of the mental lexicon. For this reason, we used specificity and concreteness measures operationalized through human judgments to investigate differences between concepts in lexical and semantic decision tasks.

Results of reaction times analyses differ between the two reported studies. In Study 2, we found that both concreteness and specificity had no effect in explaining RTs during lexical decision. This result can be explained by the relatively shallow process involved in lexical processing task, where the semantic information is less influential than lexical-level elements (Barsalou 2008; Yap et al. 2011), especially when stimuli are balanced for relevant linguistic dimensions, such as word length and word frequency. Similarly, Yap et al. (2011) demonstrated that if controlled for lexical variables, the “semantic variables explained only 2% of additional variance in lexical decision latencies” (Pexman et al. 2017). Nevertheless, a visual and qualitative inspection of the influence of word’s concreteness and specificity on predicted RTs (Fig. 1) suggest that concepts with lower specificity, i.e., general ones, require less effort to be recognized, regardless of their concreteness level. This tendency is in line with previous (Iliev and Axelrod 2017; Bolognesi and Caselli 2023) and current (Ravelli et al. 2024) research showing a positive effect of specificity over response latencies in LDT, where more general words are associated with faster response than specific words. Moreover, our data suggests that the processing disadvantage is more marked for concepts that score high in specificity and low in concreteness exhibit the slowest recognition times (i.e., abstract-specific). The present results are intriguing and merit further investigation. For instance, future studies could replicate the same paradigm with longer word presentation times to ensure a less superficial judgment.

In Study 3, we found a significant main effect of concreteness (i.e., concrete words were recognized faster than abstract ones) and a significant main effect of specificity (specific words were recognized faster than general ones) during abstract/concrete semantic categorization task. Notably, the interaction between concreteness and specificity was not significant. This demonstrates that categorical specificity has an independent significant contribution in predicting responses times in a task implying a deeper semantic processing level. These findings align with Ravelli and colleagues’ study (2024), in which the authors compared SDT’s chronometric data (Pexman et al. 2017) with specificity scores (Bolognesi and Caselli 2023) and found a negative effect of specificity: more specific words are processed faster than more general ones. Similarly, in the present semantic decision task we found that specificity and concreteness have a similar negative effect over RTs: an increase in responses times occurred during processing of words with low specificity scores and low concreteness scores. However, regardless of concreteness level, highly specific words required less effort to be recognized as concrete or abstract respectively, compared to general ones. One plausible interpretation for our findings could be that the categorization of concrete and abstract words may be affected by the cognitive effort needed to abstract a referent from a single or multiple exemplars instantiated. This hypothesis is in line with an exemplar-based theory of conceptual category representations (e.g., Nosofsky 1986; Daelemans and van den Bosch 2005; Chandler 2017), according to which speakers engaging in a semantic decision task or other kinds of semantics-based cognitive decisions (e.g., typicality judgement and exemplar production tasks, see also Heit and Barsalou 1996) are likely to mentally instantiate exemplars or referents. When confronted with a word that is quite general (e.g., *substance*) speakers might mentally instantiate more diverse exemplars of the category, before deciding whether it denotes a concrete or abstract entity, leading to longer processing times. Conversely, when presented with a more specific word (e.g., *Aspirin*) they may effortlessly instantiate a single exemplar, resulting in faster processing. This is also consistent with seminal findings conducted on concrete conceptual categories, by Rosch et al. (1976) and Rosch (1978), showing that superordinate categories require more effort to be processed and conceived compared to concept at other taxonomic levels. Interestingly, in our study this effect seems to emerge also among abstract concepts. Traditionally, specific concepts are characterized by high informativeness and low distinctiveness, whereas general concepts tend to be less informative and more distinctive (Murphy 2004). Therefore, specific concepts could activate narrow and dense representations, associated to the instantiation of a small number of precise exemplars and converging on more salient and accessible semantic information. This “high-resolution” semantic content may support categorization in semantic decision tasks by facilitating the retrieval of task-relevant information (e.g., evaluating whether a word is abstract or concrete). Overall, our studies provided pivotal insights into the distinction between concreteness and categorical specificity. Firstly, in line with Bolognesi and colleagues (2020), we confirmed the weak correlation between the two variables (*r* = 0.29). Secondly, we established their non-collinearity. Thirdly, we found their additive effect over decision latencies in a behavioral experiment, such as the semantic decision task. Lastly, although the results from the lexical decision task did not reach statistical significance, the pattern observed across the two categorization tasks revealed a consistent effect of concreteness, with more concrete concepts requiring less processing time. In contrast, the influence of specificity varied by task. When included in the model, specificity demonstrated opposite effects on reaction times across the two tasks. In the semantic decision task, words with higher specificity were processed more quickly than those with lower specificity. However, in the lexical decision task, the reverse pattern emerged. Taken together, these findings provide further support for the view that concreteness and specificity represent two distinct semantic dimensions, each contributing differently to word processing depending on task demands.

Moreover, the differences observed across categorization tasks support the possibility that the influence of semantic variables may vary depending on the nature of the task (Goh et al. 2016; see also Pexman 2012; Pexman et al. 2017). For instance, studies on *numbers of senses* (Borowsky and Masson 1996) showed dissociation effect on RTs in LDT and SDT, as pointed out by Yap et al. (2011, p. 724): “words with more senses elicited shorter lexical decision times but less accurate semantic classification responses”. Given that general words tend to have more possible senses and are applicable across a wider range of contexts compared to specific words, it is reasonable to assume that their processing may benefit from broader semantic activation. This could result in faster responses during lexical decision tasks, where deep access to a word’s meaning is not required. In contrast, semantic categorization tasks demand a deeper understanding of word meaning to make accurate classifications. In this context, the greater number of senses and the activation of multiple competing exemplars associated with general words may hinder performance. As a result, general words show a significant disadvantage compared to specific words in terms of semantic decision latencies. Specific words, by constraining the range of possible meanings, offer information that is sufficiently precise and focused to facilitate faster and more accurate semantic classification.

In contrast to the perspective proposed by Iliev and Axelrod (2017), we suggest that informativeness at the subordinate level may actually enhance performance in semantic decision tasks, as it provides more direct access to relevant conceptual information.

Furthermore, the typically low dimensionality of general concepts, as described by Lupyan and Mirman (2013), may help explain the increased cognitive effort required for their processing and the resulting delay in determining whether a concept is concrete or abstract in semantic decision tasks. General concepts often represent broader, more inclusive categories with fewer salient shared features, which may make them more difficult to evaluate quickly. Because low dimensionality is a property commonly associated with abstract concepts, the observed differences in response latencies between highly abstract but general concepts and highly abstract but specific concepts may reflect a more precise and constrained representation for the latter.

Recent findings by Rambelli and Bolognesi (2023) support this interpretation, showing that abstract-specific concepts tend to exhibit low contextual variability (Hoffman 2016)—that is, they are associated with a relatively limited range of accessible contexts. According to Contextual Availability Theory (Schwanenflugel et al. 1988), the ease with which relevant contexts can be retrieved from memory is linked to faster word processing. Therefore, integrating our results with previous research, it is reasonable to propose that the quantity and specificity of information associated with a concept—whether in terms of exemplars, meanings, features, or contexts—can result in either a processing advantage or disadvantage, depending on the task. Further research is needed to clarify the specific contribution of these informational dimensions to semantic processing.

This study has some limitations, including the absence of basic-level concepts and the use of a semantic categorization task focused specifically on the abstract–concrete distinction. However, our primary objective was to conduct a targeted comparison of words positioned at the extremes of the specificity continuum, from highly general to highly specific—an area that remains underexplored in current research. To ensure comparability with existing findings, we employed a well-established task in the study of abstract and concrete concepts (Pexman et al. 2017). Although this task emphasizes judgments of concreteness, it reliably engages in deep semantic processing. In contrast, collecting reaction time data using alternative categorization tasks, such as animacy judgments or direct specificity ratings, would have been considerably more complex due to the diversity of semantic domains in our stimuli and the inherently relational nature of specificity, which depends on comparisons between concepts.

Despite these constraints, the observed specificity effect, along with the improved model fit when specificity was included as a predictor of reaction times, highlights the importance of incorporating word specificity into research on the abstract–concrete distinction.

Furthermore, the ongoing debate on the concreteness effect could benefit from greater attention to taxonomic levels of abstraction when selecting stimuli for behavioral tasks. The identification of a specificity effect, particularly within the context of a semantic categorization task, suggests that some of the inconsistencies observed in previous studies on the concreteness effect may be due to the omission of specificity as a contributing factor to the informativeness of semantic representations. We recommend that future research on the processing of abstract and concrete concepts take specificity into account, whether through the design of new experiments or the reanalysis of existing data.

Specificity is a crucial factor in semantic access and should not be overlooked or confused with concreteness. The results of the present study provide further evidence that specificity should be considered a key variable within the framework of semantic richness. It appears to activate semantic memory automatically and to influence language processing by accounting for independent variance in decision latencies.

## Conclusions

The present study demonstrated that concreteness and categorical specificity are distinct dimensions of the abstraction process. In light of the mixed findings surrounding the concreteness effect, our work introduces evidence of a specificity effect in a semantic categorization task: specific concepts are processed more quickly than general ones. Both abstract and concrete specific concepts appear to involve more refined conceptual representations, supported by salient and focused information. In contrast, general concepts are represented through a broader set of features, situations, and category members, which introduces greater competition and increases the cognitive effort required for abstraction, ultimately leading to longer processing times.

These findings call for further investigation into the nature of the information that supports specificity and into the extent to which this specification enhances conceptual representation.

## Data Availability

Data, scripts, and results output of the three studies are stored in a public online repository on Open Science Framework: https://osf.io/j2smv/.
